# Early-Stage Ruptured Hepatocellular Carcinoma With Different Tumor Diameters: Small Tumors Have a Better Prognosis

**DOI:** 10.3389/fonc.2022.865696

**Published:** 2022-05-17

**Authors:** Feng Xia, Zhiyuan Huang, Qiao Zhang, Elijah Ndhlovu, Mingyu Zhang, Xiaoping Chen, Bixiang Zhang, Peng Zhu

**Affiliations:** ^1^Department of Hepatic Surgery, Tongji Hospital, Tongji Medical College of Huazhong University of Science and Technology, Wuhan, China; ^2^Guangdong Medical University, Zhanjiang, China; ^3^Department of Digestive Medicine, Tongji Hospital of Tongji Medical College of Huazhong University of Science and Technology, Wuhan, China

**Keywords:** ruptured hepatocellular carcinoma, BCLC stage, prognosis, tumor diameter, TNM

## Abstract

**Background and Aim:**

Ruptured hepatocellular carcinoma (rHCC) is classified as T4 according to the TNM staging system with a very poor (does not achieve expected) prognosis, which has always been controversial. This study aimed at assessing the specific impact of different tumor diameters on the posttreatment prognosis of BCLC stage 0/A rHCC patients.

**Methods:**

Data from 258 patients with BCLC stage 0/A HCC treated in our center from January 2008 to December 2017 were collected, including 143 rHCC patients and 115 patients with non-ruptured HCC (nrHCC). With the help of X-tile software, we determined the cutoff value of the tumor diameter in patients with rHCC. Using 8 cm as the cutoff, we divided rHCC patients into Small-rHCC (n = 96) and Large-rHCC (n = 47) groups, compared the prognoses of the S-rHCC and L-rHCC groups, as well as the prognoses of the two groups with the nrHCC group using the Kaplan–Meier method, and screened the prognostic factors of rHCC patients using the multivariate Cox risk model.

**Results:**

The OS of the S-rHCC group was significantly higher than that of the L-rHCC group [HR = 2.41 (1.60–3.63)], and the OS of the nrHCC group was comparable to that of the S-rHCC group (P = 0.204). In patients treated with surgery only, OS and RFS were also comparable in the S-rHCC nrHCC group. Meanwhile, multivariate Cox regression analysis revealed that alpha-fetoprotein (AFP), alkaline phosphatase (ALP), and the main method of treatment were also prognostic factors for OS in patients with rHCC.

**Conclusions:**

Ruptured HCC with a relatively small diameter (≤8 cm) can also achieve the same prognosis as nrHCC patients after aggressive treatment. It is also not recommended to include all patients with rHCC in stage T4.

## Introduction

The incidence of ruptured hepatocellular carcinoma (rHCC) is gradually increasing, reaching as high as 10%–15% in some parts of Asia, and hepatocellular carcinoma (HCC) is generally considered to have an inferior prognosis once it ruptures (1, 2). At the same time, in TNM staging, rHCC is included in T4. Some researchers, such as Jitrapa Kerdsuknirun (3), believe that once rupture occurs, it will lead to a poor prognosis regardless of treatment. However, some patients with rHCC can also achieve a better prognosis after different treatments. Darren W. Chua et al. (4) used the propensity score matching (PSM) method to compare the prognosis of ruptured and unruptured tumors after surgery. They believed that the prognosis of the two groups was equivalent; Luciano Tarantino et al. (5) also believed that for some patients with rHCC who have better liver function compensation, timely surgical treatment could obtain a better prognosis. Therefore, we can see that a relatively better prognosis can be achieved after treatment in some selected patients with rHCC. Tumor diameter is an essential factor affecting the prognosis of patients; Hiroyuki Kirikoshi (6) concluded that maximum tumor size is an independent prognostic factor affecting the survival of patients with rHCC.

Our study investigated the effect of different tumor diameters on the posttreatment outcome of patients with rHCC. The rHCC results were compared with nrHCC to determine the impact of rupture on the prognosis of HCC patients.

## Materials and methods

### Patient Selection

We carefully collected 258 patients with BCLC stage 0/A tumors in our hospital from January 2008 to December 2017, including 143 patients with ruptured HCC and 115 patients with non-ruptured HCC; the patient screening process is described in **Supplementary Figure 1**. By utilizing X-tile software (7), the maximum tumor diameter cutoff of rHCC patients was determined to be 7.9 cm. To make it more convenient for clinicians, 8.0 cm was defined as the cutoff value. The cutoff value was then applied to divide rHCC patients into S-rHCC and L-rHCC groups. The patients received surgical resection, TACE, or conservative treatment. The inclusion criteria were as follows. (1) Tumor rupture was determined by contrast-enhanced CT or MRI. (2) Patients who underwent surgical treatment were determined to have HCC by pathology report; those who underwent other treatments were determined to have HCC by contrast-enhanced CT and/or MRI combined with a medical history and serum alpha-fetoprotein levels. (3) The BCLC 0/A stage was determined by a combination of experienced clinicians and radiologists. (4) There should be good liver function (Child–Pugh A/B). (5) Those who underwent surgical treatment were R0 resected (R0 resection means no residual tumor cells were found at the microscopic resection margin by a pathologist). (6) All tumors were first discovered tumors. (7) No other antitumor treatment was received before admission. The exclusion criteria were (1) metastasis of liver cancer, (2) presence of macrovascular invasion, (3) diagnosis of non-HCC by two experienced pathologists, (4) incomplete clinical data, and (5) incomplete follow-up information. This retrospective study was reviewed and approved by the Ethics Committee of Wuhan Tongji Hospital. All patients signed the consent forms.

### Treatment Selection

All patients with ruptured HCC would receive appropriate treatment such as intravenous fluids/transfusions after admission. TACE or conservative treatment is the first choice for patients with unstable blood flow caused by acute abdominal hemorrhage. If TACE or conservative treatment is unsatisfactory, surgical treatment will be performed for patients with preserved liver function; if bleeding is stopped spontaneously after TACE or conservative treatment, a two-stage hepatectomy will be performed. TACE is the therapy of choice for patients who cannot undergo surgical treatment. For patients undergoing surgical treatment, the liver function screening criteria included child class A/B, preoperative evaluation of residual liver volume needed to be greater than 40% of the standard liver volume, and ICG-R15 of **≤**45%; TACE was performed by inserting a microcatheter into the artery supplying the tumor after hepatic angiography, injecting chemotherapeutic drugs, and embolizing the artery with a gelatin sponge. All surgical treatments were open hepatectomy.

All treatment recommendations were based on the clinical judgment of experienced physicians in our center (Department of Hepatobiliary Surgery, Gastroenterology, and Ultrasound Imaging). The treatment choice of the patients or their families is considered when determining the type of treatment modality. Preoperative assessment was performed thoroughly before deciding on methods of liver resection. The resectability of the primary tumor and metastases was assessed by hospital ultrasound.

### Identification of Pathological and Clinical Variables

Sixteen variables that may affect the prognosis of HCC were collected for statistical analysis, including related variables such as patients**’** basic condition, liver and basic tumor characteristics, and pathological factors, such as age, gender, tumor max diameter, tumor number, pretherapy alanine aminotransferase (ALT), pretherapy alpha-fetoprotein (AFP), pretherapy albumin (ALB), pretherapy alkaline phosphatase (ALP), pretherapy aspartate aminotransferase (AST), pretherapy gamma-glutamyl transferase (GGT), HBsAg, Edmondson–Steiner grade, microvascular invasion (MVI), and satellite foci. Two experienced pathologists completed the pathology report.

### Follow-Up

Follow-up for all patients was performed at specified intervals. The follow-up was performed every 3 months within the first year after discharge, and every half a year after the first year. Imaging examinations, such as enhanced CT and abdominal MRI, as well as laboratory examinations, such as liver function, AFP, and other tumor markers, were performed at follow-up. For surgical patients, overall survival (OS) is defined as the time from the first day after surgery to death, and recurrence-free survival (RFS) is defined as the time from the first day after surgery to the first radiographic finding of neoplasm or metastasis. For patients receiving TACE or conservative treatment, OS is defined as the first day after admission to receive treatment to death. The follow-up period was up to September 30, 2021.

### X-Tile Analysis

The X-tile analysis developed at Yale University was performed to assess tumor diameter; this was expressed as an optimized cutoff point based on overall survival outcome. Statistical significance was assessed by the standard log-rank method using a cutoff score of 143 patients with P values from a lookup table (7).

### Propensity Score Matching Analysis

Some confounding factors can lead to inaccuracy of the results. In this study, there were three variables with statistical differences in rHCC, which were alpha-fetoprotein (AFP), aspartate aminotransferase (AST), and gamma-glutamyltransferase (GGT). We included them in the PSM model to balance the baseline. We performed 1:1 matching using SPSS 25.0. We chose a 0.1 caliper width so that an optimal trade-off can be obtained.

### Data Analysis

The data of this study were binary variables. The chi-squared test or Fisher’s exact test was used to compare categorical variables. The Kaplan–Meier method was used for comparison of the OS and RFS of the patients. The log-rank test was used for comparison of survival rates. Univariate and multivariate analyses were performed for OS and RFS after hepatectomy using the Cox proportional hazards model. Variables with P < 0.05 in the univariate analysis were included in the multivariate regression analysis.

All data analysis was performed by SPSS 25.0 and R software (version 4.0.5) and graphically plotted using R software (version4.0.5); the cutoff value of the maximum tumor diameter was obtained by X-tile software.

## Results

### Selection of the Cutoff Value for the Maximum Diameter of rHCC

**Figure 1:**
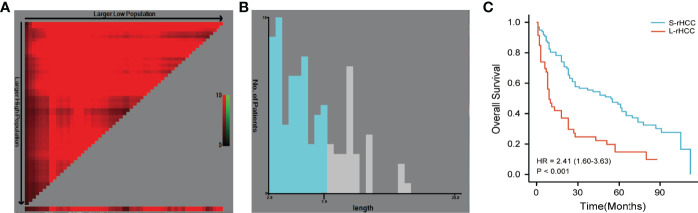
The cutoff value determined by X-tile software was 7.9 cm **(A, B)**. The cutoff value of 8.0 cm was selected to divide patients into S-rHCC and L-rHCC groups. There was a significant difference in OS between the two groups [HR = 2.41 (1.60–3.63), P < 0.001] **(C)**.

We first used X-tile software developed at Yale University to select the optimal cutoff value based on overall survival (OS), and from **Figures 1A, B**, it can be seen that at cutoff = 7.9** cm**, the P-value of the two groups was the smallest and the two curves were the most distinguished. In order to make it more convenient for clinicians, we chose 8** cm** as the cutoff value. At the same time, we also selected the theory of the value corresponding to the maximum Youden index based on sensitivity and specificity. The receiver operating characteristic curve (ROC) was used to determine the tumor diameter value for predicting the overall survival rate. The optimal cutoff value was 7.1 cm. We compared the discrimination of tumor diameter at two different cutoff values by the time-dependent ROC curve. The results showed that the AUC with a cutoff value of 8.0 cm was higher than the AUC with a cutoff value of 7.1 cm in 1-, 3-, and 5-year OS, and 8.0** cm** was still the selected cutoff value.

### The Basic Characteristic of rHCC Patients in BCLC Stage 0/A

The patients with rHCC were divided into S-rHCC group (<8 cm) and L-rHCC (>8 cm) group. Most of them were men in the two groups. In the S-rHCC group, 80 patients (83.3%) received surgical treatment, 14 patients (14.6%) received TACE treatment, and 2 patients (2.1%) received conservative treatment; in the L-rHCC group, 30 patients (63.8%) received surgical treatment, 16 patients (34.0%) received TACE treatment, and 1 patient (2.1%) received conservative treatment. There was a statistically significant difference in differentiation between the two groups (P < 0.05), and the serum levels of AST and GGT in the two groups and the remaining variables were balanced **(** **Table 1****).**

**Table 1:** Baseline characteristics of patients with the BCLC 0/A stage in the small ruptured HCC group (S-rHCC) and large ruptured HCC group (L-rHCC) (*n* = 143).

		S-rHCC (*n* = 96)	L-rHCC (*n* = 47)	P-value
Gender				1.000
	Male	87 (90.6)	43 (91.5)	
	Female	9 (9.4)	4 (8.5)	
Age				0.498
	≤60 y	80 (83.3)	37 (78.7)	
	>60 y	16 (16.7)	10 (21.3)	
Tumor number				0.551
	Single	93 (96.9)	47 (100.0)	
	Multiple	3 (3.1)	0 (0.0)	
Tumor location				0.231
	Left	22 (22.9)	12 (25.5)	
	Right	46 (47.9)	21 (44.7)	
	Middle	28 (29.2)	14 (29.8)	
AFP				0.477
	≤400 ng/ml	48 (50.0)	27 (57.4)	
	>400 ng/ml	48 (50.0)	20 (42.6)	
Child–Pugh grade				0.834
	A	75 (78.1)	36 (76.6)	
	B	21 (21.9)	11 (23.4)	
MELD score				0.384
	6–8	38 (39.6)	19 (40.4)	
	9–12	36 (37.5)	18 (38.3)	
	≥13	22 (22.9)	10 (21.3)	
Cirrhosis				0.204
	No	75 (78.1)	33 (70.2)	
	Yes	21 (21.9)	14 (29.8)	
Differentiation grade				0.033
	Edmondson–Steiner I/II	50 (52.1)	18 (38.3)	
	Edmondson–Steiner III/IV	30 (31.3)	12 (25.5)	
	Unknown	16 (16.7)	17 (36.2)	
MVI				0.007
	No	60 (62.5)	17 (36.2)	
	Yes	20 (20.8)	13 (27.7)	
	Unknown	16 (16.7)	17 (36.2)	
Satellite foci				0.004
	No	48 (50.0)	11 (23.4)	
	Yes	32 (33.3)	19 (40.4)	
	Unknown	16 (16.7)	17 (36.2)	
HBsAg				0.331
	No	17 (17.7)	5 (10.6)	
	Yes	79 (82.3)	42 (89.4)	
Prothrombin time (s)		15.2 (14.3–17.8)	16.1 (14.6–18.9)	0.485
Platelet (× 10^9^/l)		149.5 (122.3–193.0)	148.4 (113.3–194.0)	0.886
ALB				0.721
	≤35 g/l	55 (57.3)	25 (53.2)	
	>35 g/l	41 (42.7)	22 (46.8)	
ALT				0.036
	≤50 U/l	79 (82.3)	31 (66.0)	
	>50 U/l	17 (17.1)	16 (34.0)	
AST				0.010
	≤40 U/l	67 (69.8)	22 (46.8)	
	>40 U/l	29 (30.2)	25 (53.2)	
ALP				0.882
	≤100 U/l	76 (79.2)	37 (78.7)	
	>100 U/l	20 (20.8)	10 (21.3)	
GGT				0.041
	≤60 U/l	66 (68.8)	24 (51.1)	
	>60 U/l	30 (31.3)	23 (48.9)	
Main treatment				0.027
	Hepatectomy	80 (83.3)	30 (63.8)	
	TACE	14 (14.6)	16 (34.0)	
	Conservative	2 (2.1)	1 (2.1)	

BCLC, Barcelona Clinic Liver Cancer; AFP, alpha-fetoprotein; HCC, hepatocellular carcinoma; rHCC, ruptured hepatocellular carcinoma; TACE, transcatheter arterial chemoembolization; MELD, model for end-stage liver disease; MVI, microvascular invasion; HBsAg, hepatitis B surface antigen; ALB, albumin; ALT, alanine aminotransferase; AST, aspartate aminotransferase; ALP, alkaline phosphatase; GGT, γ-glutamyl transpeptidase.

### Prognosis in the S-rHCC Group and L-rHCC Group in the BCLC Stage 0/A

As shown in **Figure 1C**, there was a significant difference in OS between the two groups [HR = 2.41 (1.60–3.63)]. In S-rHCC, the 1-, 3-, and 5-year overall survival rates were 81.2%, 56.1%, and 46.7%, and the median overall survival time was 55.0 months; in L-rHCC, the 1-, 3-, and 5-year overall survival rates were 43.5%, 24.2%, and 14.5%, and the median overall survival time was 9.0 months.

### The S-rHCC Group and L-rHCC Group Were Sub-Analyzed

There were differences between the two groups in MVI and satellite foci variables. The subgroup analysis was performed according to the positive and negative results of MVI and satellite foci. From **Supplementary Figure 2**, whether the MVI was positive or the satellite foci were positive, the prognoses of the S-rHCC and L-rHCC groups were statistically different **(** **Supplementary Figure 2****).**

### Prognostic Factors for Survival in BCLC Stage 0/A rHCC Patients

By univariate and multivariate regression analysis, tumor max diameter [HR = 1.795 (1.152–2.798)], AFP [HR = 1.955 (1.294–2.955)], ALP [HR = 2.584 (1.488–4.486)], and different treatment modalities were prognostic factors for BCLC stage 0/A rHCC patients. There were three treatments: conservative treatment used as a control, hepatectomy [HR = 0.023 (0.005–0.106)], and TACE [HR = 0.056 (0.012–0.257)] **(** **Table 2****).**

**Table 2:** Univariate and multivariate analyses of overall survival in BCLC 0/A stage rHCC patients.

	Univariate analysis			Multivariate analysis		
	P	HR	95% confidence interval	P	HR	95% confidence interval
**Gender** Male/female	0.299	1.033	0.676–1.578			
**Age** ≥60 y/<60 y	0.192	0.662	0.356–1.231			
**Tumor max diameter** ≥8 cm/<8 cm	**<0.001**	2.048	1.368–3.065	0.010	1.795	1.152–2.798
**Tumor location** Unilateral/middle	0.056	0.925	0.745–1.013			
**AFP** ≥400 ng/ml/<400 ng/ml	**0.024**	1.619	1.064–2.464	0.001	1.955	1.294–2.955
**Cirrhosis** Yes/no	**0.028**	1.513	1.308–1.935			
**HBsAg** Yes/no	0.100	1.812	0.892–3.681			
**Prothrombin time** Per s	0.875	1.003	0.96–1.011			
**ALB** ≥35 g/l/<35 g/l	0.079	0.656	0.411–1.049			
**ALT** ≥50 U/l/<50 U/l	0.072	0.554	0.291–1.055			
**AST** ≥40 U/l/<40 U/l	0.360	1.335	0.719–2.479			
**ALP** ≥100 U/l/<100 U/l	**<0.001**	3.539	1.920–6.523	0.001	2.584	1.488–4.486
**GGT** ≥60 U/l/<60 U/l	0.676	0.892	0.356–1.231			
**Main treatment**	**<0.001**			<0.001		
Hepatectomy/conservative	**<0.001**	0.028	0.006–0.138	<0.001	0.023	0.005–0.106
TACE/conservative	**0.001**	0.066	0.014–0.320	<0.001	0.056	0.012–0.257

BCLC, Barcelona Clinic Liver Cancer; AFP, alpha-fetoprotein; HCC, hepatocellular carcinoma; rHCC, ruptured hepatocellular carcinoma; TACE, transcatheter arterial chemoembolization; MVI, microvascular invasion; HBsAg, hepatitis B surface antigen; ALBI, albumin–bilirubin grade; ALT, alanine aminotransferase; AST, aspartate aminotransferase; ALP, alkaline phosphatase; GGT, γ-glutamyl transpeptidase. Bold means statistically significant in Univariate cox regression analysis.

### The Basic Characteristics of HCC Patients in BCLC Stage 0/A and Baseline Table of nrHCC and rHCC After PSM

A total of 115 patients with unruptured HCC were included in the study. The serum levels of AFP, AST, and GGT were statistically different between the two groups in the nrHCC group, but there was no statistical difference in the other variables. The patients in the nrHCC group also had three different treatment methods, hepatectomy, TACE, and conservative treatment, of which 110 (76.9%) received hepatectomy, 30 (21.0%) received TACE, and 3 (2.1%) received conservative treatment **(** **Table 3****).** After PSM, all variables in the nrHCC group were balanced, without any statistically significant differences **(** **Supplementary Table 1****).**

**Table 3:** Baseline characteristics of nrHCC and rHCC patients in BCLC 0/A stage (n = 258).

		rHCC (n = 143)	nrHCC (n = 115)	P-value
Gender				1.000
	Male	130 (90.9)	104 (90.4)	
	Female	13 (9.1)	111 (9.6)	
Age				0.052
	≤60 y	117 (81.8)	104 (90.4)	
	>60 y	26 (18.2)	11 (9.6)	
Tumor number				0.745
	Single	140 (97.9)	112 (97.3)	
	Multiple	3 (2.1)	3 (2.7)	
AFP				<0.001
	≤400 ng/ml	75 (52.4)	28 (24.3)	
	>400 ng/ml	68 (47.6)	87 (75.7)	
Child–Pugh				0.090
	A	111 (77.6)	78 (67.8)	
	B	32 (22.4)	37 (32.2)	
Cirrhosis				0.104
	No	111 (77.6)	80 (69.6)	
	Yes	32 (22.4)	35 (30.4)	
HBsAg				0.271
	No	22 (15.4)	12 (10.4)	
	Yes	121 (84.6)	103 (89.6)	
ALB				1.000
	≤35 g/l	80 (55.9)	64 (55.7)	
	>35 g/l	63 (44.1)	51 (44.3)	
ALT				1.000
	≤50 U/l	110 (76.9)	88 (76.5)	
	>50 U/l	33 (23.1)	27 (23.5)	
AST				0.008
	≤40 U/l	89 (62.2)	52 (45.2)	
	>40 U/l	54 (37.8)	63 (54.8)	
ALP				0.190
	≤100 U/l	113 (78.9)	82 (71.3)	
	>100 U/l	30 (21.1)	33 (28.7)	
GGT				0.001
	≤60 U/l	89 (62.7)	48 (41.7)	
	>60 U/l	53 (37.3)	67 (58.3)	
Main treatment				0.544
	Hepatectomy	110 (76.9)	82 (71.3)	
	TACE	30 (21.0)	29 (25.2)	
	Conservative	3 (2.1)	4 (3.5)	

BCLC, Barcelona Clinic Liver Cancer; AFP, alpha-fetoprotein; HCC, hepatocellular carcinoma; rHCC, ruptured hepatocellular carcinoma; TACE, transcatheter arterial chemoembolization; HBsAg, hepatitis B surface antigen; ALB, albumin; ALT, alanine aminotransferase; AST, aspartate aminotransferase; ALP, alkaline phosphatase; GGT, γ-glutamyl transpeptidase.

### Prognosis Among the Three Groups of S-rHCC, L-rHCC, and nrHCC

**Figure 2:**
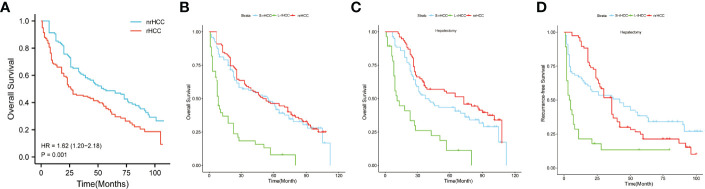
There was a significant difference in OS between ruptured HCC and non-ruptured HCC patients [HR = (1.62 (1.20–2.18), P = 0.001] **(A, B)** represents all treatment means; **(C)** represents patients treated with surgery only (OS); **(D)** represents patients treated with surgery only (RFS).

All patients were in BCLC stage 0/A, and it can be seen from **Figure 2A** that the prognosis of rHCC was worse than that of nrHCC [HR = 1.62 (1.20–2.18) P = 0.001]. **Supplementary Figure 3** shows that after PSM, the prognosis of rHCC was still worse than that of nrHCC [HR = 2.02 (1.38–2.97) P < 0.001] **(** **Supplementary Figure 3****)**. As shown in **Figure 2B**, the OS of nrHCC patients was comparable to that of S-rHCC (P = 0.204), with 1-, 3-, and 5-year OS rates of 90.4%, 60.9%, and 48.7%, and a median survival time of 55.0 months. We compared the prognosis of the three groups treated with surgery only, and in **Figure 2C**, the OS of the nrHCC and S-rHCC groups treated with surgery remained comparable (P = 0.053). The 1-, 3-, and 5-year OS rates for nrHCC patients were 98.8%, 75.5%, and 60.6%, and the median survival time was 76.0 months. The 1-, 3-, and 5-year OS rates were 91.2%, 59.5%, and 48.1%, and the median survival time was 57.0 months in the S-rHCC group. In **Figure 2D**, the RFS for patients in the S-rHCC group who only received surgical treatment was comparable to the nrHCC patients who received surgical treatment alone (P = 0.835), and the 1-, 3-, and 5-year RFS rates were 93.9%, 53.5%, and 31.6%, and the median time to recurrence was 36.0 months in the nrHCC group; the 1-, 3-, and 5-year RFS rates were 66.2%, 51.5%, and 38.0%, and the median time to recurrence was 42.0 months in the S-rHCC group.

### Prognostic Factors for Survival in All Patients With BCLC Stage 0/A HCC

After univariate and multivariate analyses, using S-rHCC as reference, we found that in the prognoses of the three groups of S-rHCC, L-rHCC, and nrHCC, HR = 3.235 [2.167–4.830] in the L-rHCC group (P < 0.001) and HR = 1.380 [0.982–1.939] in the nr-rHCC group (P = 0.063). Moreover, for the treatment modality, HR = 2.430 [1.717–3.440] for hepatectomy with TACE as a reference (P < 0.001) **(** **Table 4****).**

**Table 4:** Univariate and multivariate analyses of overall survival among S-rHCC, L-rHCC, and nrHCC in the BCLC 0/A stage.

	Univariate analysis			Multivariate analysis		
	P	HR	95% confidence interval	P	HR	95% confidence interval
**Gender** Male/female	0.733	0.921	0.574–1.478			
**Age** ≥60 y/<60 y	0.768	0.940	0.623–1.418			
**AFP** ≥400 ng/ml/<400 ng/ml	**<0.001**	1.864	1.395–2.491	0.008	1.631	1.135–2.345
**Cirrhosis** Yes/no	**0.022**	1.446	1.055–1.982	0.473		
**HBsAg** Yes/no	**0.028**	1.677	1.056–2.663	0.442		
**ALB** ≥35 g/l/<35 g/l	0.146	0.815	0.619–1.073			
**ALT** ≥50 U/l/<50 U/l	0.350	1.165	0.846–1.603			
**AST** ≥40 U/l/<40 U/l	**0.003**	1.516	1.154–1.991	0.187		
**ALP** ≥100 U/l/<100 U/l	**<0.001**	2.367	1.735–3.228	0.076		
**GGT** ≥60 U/l/<60 U/l	**<0.001**	1.965	1.493–2.585	0.046	1.566	1.008–2.432
**Group**	**<0.001**			<0.001		
L-rHCC/S-rHCC	**<0.001**	2.813	1.904–4.156	<0.001	3.235	2.167–4.830
nrHCC/S-rHCC	**0.269**	1.206	0.865–1.680	0.063	1.380	0.982–1.939
**Main treatment**						
Hepatectomy/TACE	**<0.001**	3.211	2.348–4.392	<0.001	2.430	1.717–3.440

BCLC, Barcelona Clinic Liver Cancer; AFP, alpha-fetoprotein; HCC, hepatocellular carcinoma; rHCC, ruptured hepatocellular carcinoma; TACE, transcatheter arterial chemoembolization; MVI, microvascular invasion; HBsAg, hepatitis B surface antigen; ALBI, albumin–bilirubin grade; ALT, alanine aminotransferase; AST, aspartate aminotransferase; ALP, alkaline phosphatase; GGT, γ-glutamyl transpeptidase. Bold means statistically significant in Univariate cox regression analysis.

## Discussion

Rupture of hepatocellular carcinoma is considered a severe complication (8–11), and once it occurs, the prognosis may be less than optimal even with corresponding treatment. At the same time, ruptured HCC was directly classified into T4 according to the latest TNM system (12). However, in practical clinical work, if rHCC patients are treated aggressively, a relatively good result can also be achieved, and we believe it is not appropriate to classify ruptured HCC patients directly into T4. Chan W. H (13). found that the prognosis of patients with non-ruptured stage T4 rHCC was much worse than that of patients with rupture by comparing T4 non-rupture patients with ruptured patients. At the same time, some recent studies also have many contradictions. Some researchers found that rupture does not affect the prognosis of patients through PSM matching (4), while some researchers (8, 9, 14) believed that the rupture will significantly deteriorate the prognosis of patients. However, there is still no staging system specifically for patients with rHCC, and no investigators have stratified the management of patients with rHCC. In this study, we stratified patients with BCLC stage A rHCC using the basic characteristics of tumor diameter, and we found that the prognosis of patients with small-diameter rHCC (<8.0 cm) was comparable to that of non-ruptured HCC at the same BCLC-A stage, thus indicating that not all rHCC should be considered to belong to stage T4. Some patients with rHCC can still achieve a good prognosis with aggressive treatment.

Tumor diameter plays a vital role in the prognosis of patients. Our multivariate analysis also clearly indicated the importance of tumor diameter. In the previous literature, Bing Yan et al. (15). used the SEER database to divide the tumor diameter into three groups, and there was a significant relationship between large tumor diameter and distant metastasis of HCC. Yanyan Cao et al. (16). divided the tumor diameter into three groups by the decision tree model and found that the prognosis of TACE combined with RFA treatment was worse in the group with a diameter >4.8 cm than in the other groups; Timothy M. Pawlik et al. (17) found that the higher the tumor diameter, the higher the proportion of microscopic vascular invasion, and the relatively worse the tumor differentiation. Wei-Ju Huang et al. (18) concluded that the T stage should be reclassified according to different tumor diameters, and those with a diameter of less than 3 cm should be classified into T1a, and those with a diameter of more than 3 cm should be classified into T1b. In our study, we first found the optimal cutoff value using X-tile, which was set at 8.0 cm. At this cutoff value, the prognosis of patients in different diameter groups had the most significant difference. The OS difference was 46.0 months between patients with L-rHCC and those with S-rHCC. However, the OS of patients with S-rHCC was similar to that of patients with nrHCC, not only in patients receiving different treatment methods but also in patients undergoing hepatectomy. The OS and RFS of patients with S-rHCC were similar to those with nrHCC. Therefore, based on this result, we believe that a proportion of rHCC patients screened by diameter can also achieve a similar prognosis to nrHCC patients after appropriate treatment.

This study’s main treatment methods for rHCC patients were surgery, TACE, and conservative treatment (1, 9, 19, 20). At present, the surgical treatment of choice for rHCC patients is basically open surgery. Even though some authors such as B. H. Lang et al. (21) believe that laparoscopy can avoid unnecessary laparotomy, from a clinical point of view, patients with rHCC have severe abdominal adhesions and conversion to open surgery is usually required halfway through the operation when a laparoscopic approach is chosen. At the same time, due to the particularity of rHCC, the visual field of laparotomy is better, and the condition of the entire abdominal cavity can be better observed. The advantages and disadvantages of rHCC treatment methods have also been controversial. Young-Joo Jin et al. (22) believed that surgery and TAE were superior to supportive treatment, and the prognosis of surgical treatment was better than TAE. Hanteng Yang et al. (19) suggested that hepatectomy was recommended for rHCC patients with better liver function, and TAE was recommended for rHCC patients with poor liver function. Wei Zhang et al. (23) believed that surgical resection was the preferred regimen for patients with resectable rHCC. In unresectable rHCC, TACE was more effective than conservative treatment. Similarly, our study concluded that surgical treatment is better than TACE or conservative treatment in early-stage rHCC (BCLC stage 0/A). With conservative treatment as a reference, the hazard ratio for surgical treatment was 0.023 [0.005–0.106] and it was 0.056 [0.012–0.257] for TACE. Staged hepatectomy is currently considered a standard modality for treating rHCC (10, 24–26), that is, first-stage TAE hemostasis, followed by second-stage hepatectomy when patients are hemodynamically stable. Previous studies (25, 26) have compared staged hepatectomy with emergency hepatectomy, and it is believed that it has a better prognosis than emergency hepatectomy. In addition to these three treatment modalities, Maria Baimas-George et al. (27) used laparoscopic microwave ablation and washout to treat rHCC, and patients also achieved a better prognosis. They could achieve hemostasis and bring about a reduction in the risk of local tumors metastasizing to the peritoneum. K. K. Ng et al. (28) also reported a case of rHCC treated by radiofrequency ablation (RFA) and had a better prognosis.

This study is also the first to refine rHCC, using the recognized prognostic factor of tumor diameter as the division point and using 8.0 cm as the cutoff value. The cutoff value used was not the same as the cutoff value used in previous studies. The value with the most significant difference was calculated using the X-tile software (7), and because the tumor diameter was one of the risk factors for rupture (3), the diameter was generally larger in patients with rHCC. We found that the prognosis of S-rHCC patients was much better than that of L-rHCC and comparable to the prognosis of the nrHCC group. We can see that the S-rHCC group accounted for 67% of all patients in the rHCC group, which also indicates that in early-stage rHCC, most patients can obtain the same prognosis as nrHCC patients through active treatment, so we suggest that rHCC should not be entirely included in T4. Previously, T. Aoki et al. (29) recommended increasing the T stage by 0.5 to 2.0 stages specifically for patients with rHCC. In the AJCC 8th edition (30), all patients with ruptured liver cancer were included in T4, which was also staged as IIIb or IIIc. Professor Albert C. Y. Chan et al. (31) stated that inclusion of all rHCC in T4 overestimates the severity of the rupture, and some patient data in the SEER database showed that patients with stage IIIb or IIIc HCC had a median survival time of fewer than 20 months, which is much worse than the median survival time of our S-rHCC group.

This study has some limitations and shortcomings. First, this study is a retrospective study with patient selection bias (but PSM and strict inclusion criteria were used in our study to minimize systematic errors), and second, the sample size was relatively small, and the population in this study was from areas with a high incidence of hepatitis B, which is different from the background in other regions such as Europe. There is no database from Europe or the United States used as a validation for our study. Third, only three treatment modalities for nrHCC patients were included. Finally, the data in this article originated from a single center, which has some limitations compared to data from multiple centers.

In conclusion, through stratifying rHCC patients by the essential characteristic of tumor diameter, we found that the S-rHCC group could achieve the same prognosis as the nrHCC group after appropriate treatment, bringing confidence in the treatment of rHCC.

## Data Availability Statement

The raw data supporting the conclusions of this article will be made available by the authors, without undue reservation.

## Ethics Statement

The studies involving human participants were reviewed and approved by the Ethics Committee of Wuhan Tongji Hospital. The patients/participants provided their written informed consent to participate in this study.

## Author Contributions

FX wrote the paper. ZH and PZ provided the ideas. QZ, BZ, XC, and MZ analyzed the data. PZ, EN, and XC reviewed and edited the manuscript. All authors contributed to the article and approved the submitted version.

## Funding

The research is funded by the (1) Natural Science Foundation of Hubei Province [2019CFB433], (2) Hengrui Hepatobiliary and Pancreatic Malignant Tumor Research Fund-Youth Research Fund [CXPJJH11800001-2018306], (3) Key Project of Science and Technology in Hubei Province [2018ACA137], and (4) General Project of Health Commission of Hubei Province [WJ2021M108]. Open-access publication fees were received from Tongji hospital for this study.

## Conflict of Interest

The authors declare that the research was conducted in the absence of any commercial or financial relationships that could be construed as a potential conflict of interest.

## Publisher’s Note

All claims expressed in this article are solely those of the authors and do not necessarily represent those of their affiliated organizations, or those of the publisher, the editors and the reviewers. Any product that may be evaluated in this article, or claim that may be made by its manufacturer, is not guaranteed or endorsed by the publisher.

## Supplementary Material

Supplementary Figure 1 Screening process for patients with RHCC and nrHCC.

Supplementary Figure 2 Survival curves of overall survival in the S-rHCC group versus the L-rHCC group in different subgroups. A and B represent patients with MVI (-) and MVI (+), respectively; C and D represent patients with Satellite (-) and Satellite (+), respectively.

Supplementary Figure 3 Survival curves for overall survival in the nrHCC versus rHCC groups after PSM.

## Abbreviations

HCC, hepatocellular carcinoma; rHCC, ruptured hepatocellular carcinoma; nrHCC, non-ruptured hepatocellular carcinoma; PSM, propensity score matching; ICG-R15, indocyanine green 15 min after administration; SEER: Surveillance, Epidemiology, and End Results; TAE, transcatheter arterial embolization; BCLC, Barcelona Clinic Liver Cancer; AFP, alpha-fetoprotein; TACE, transcatheter arterial chemoembolization; OS, overall survival; RFS, recurrence-free survival; MVI, microvascular invasion; RFA, radiofrequency ablation; HBsAg, hepatitis B surface antigen; ALB, albumin; ALT, alanine aminotransferase; AST, aspartate aminotransferase; ALP, alkaline phosphatase; GGT, γ-glutamyl transpeptidase.
