# Supplementary figures and images for: Early-Stage Ruptured Hepatocellular Carcinoma With Different Tumor Diameters: Small Tumors Have a Better Prognosis
Source: Front Oncol. 2022 May 17;12:865696. doi: 10.3389/fonc.2022.865696 (PMC9152538; doi:10.3389/fonc.2022.865696)

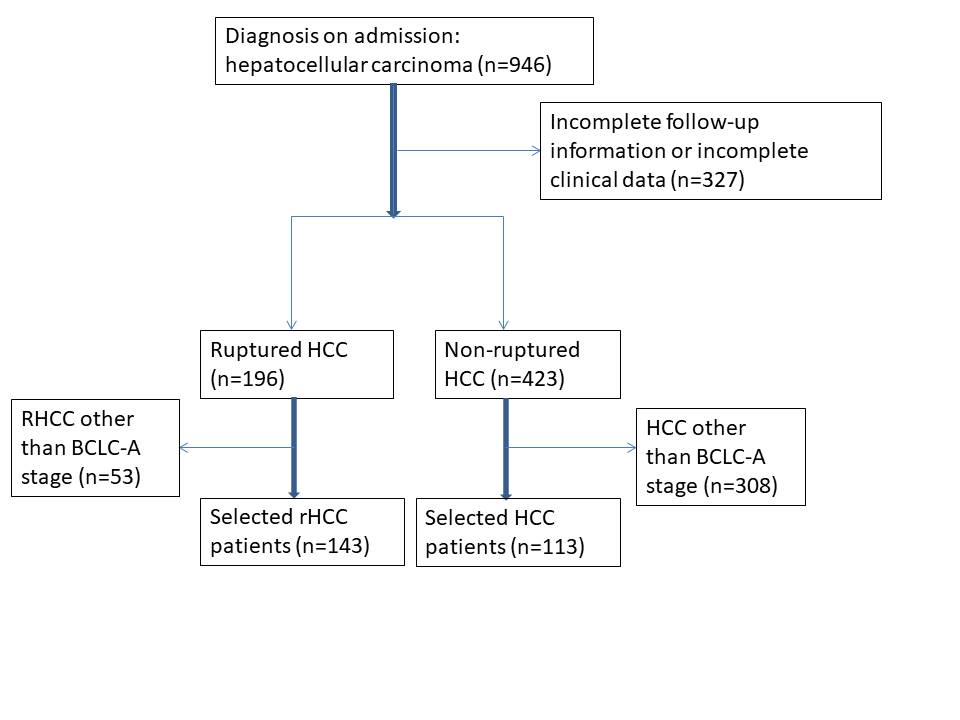

Supplement: Supplementary Figure 1 — Screening process for patients with RHCC and nrHCC. [file Image_1.jpg]

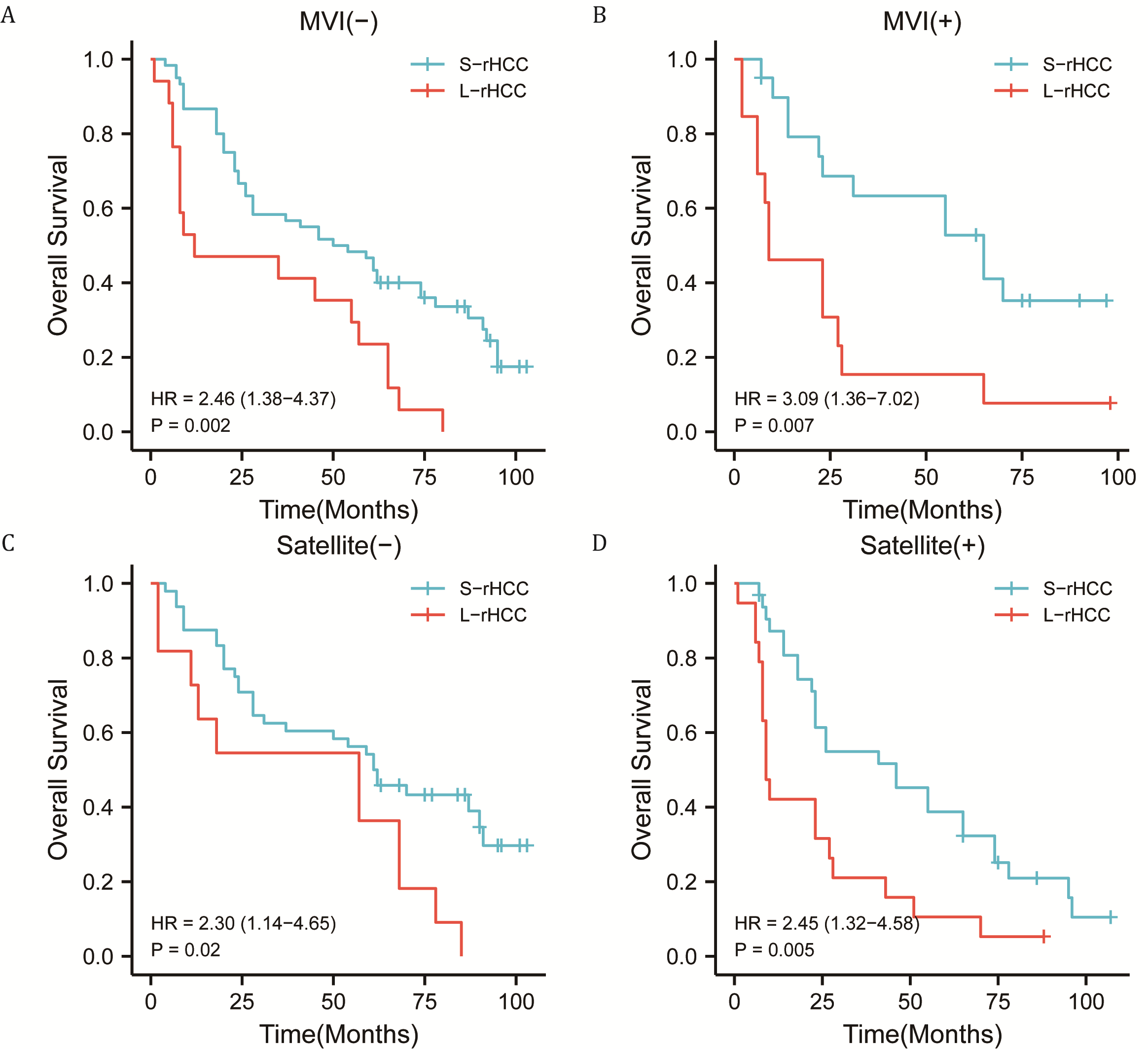

Supplement: Supplementary Figure 2 — Survival curves of overall survival in the S-rHCC group versus the L-rHCC group in different subgroups. A and B represent patients with MVI (-) and MVI (+), respectively; C and D represent patients with Satellite (-) and Satellite (+), respectively. [file Image_2.png]

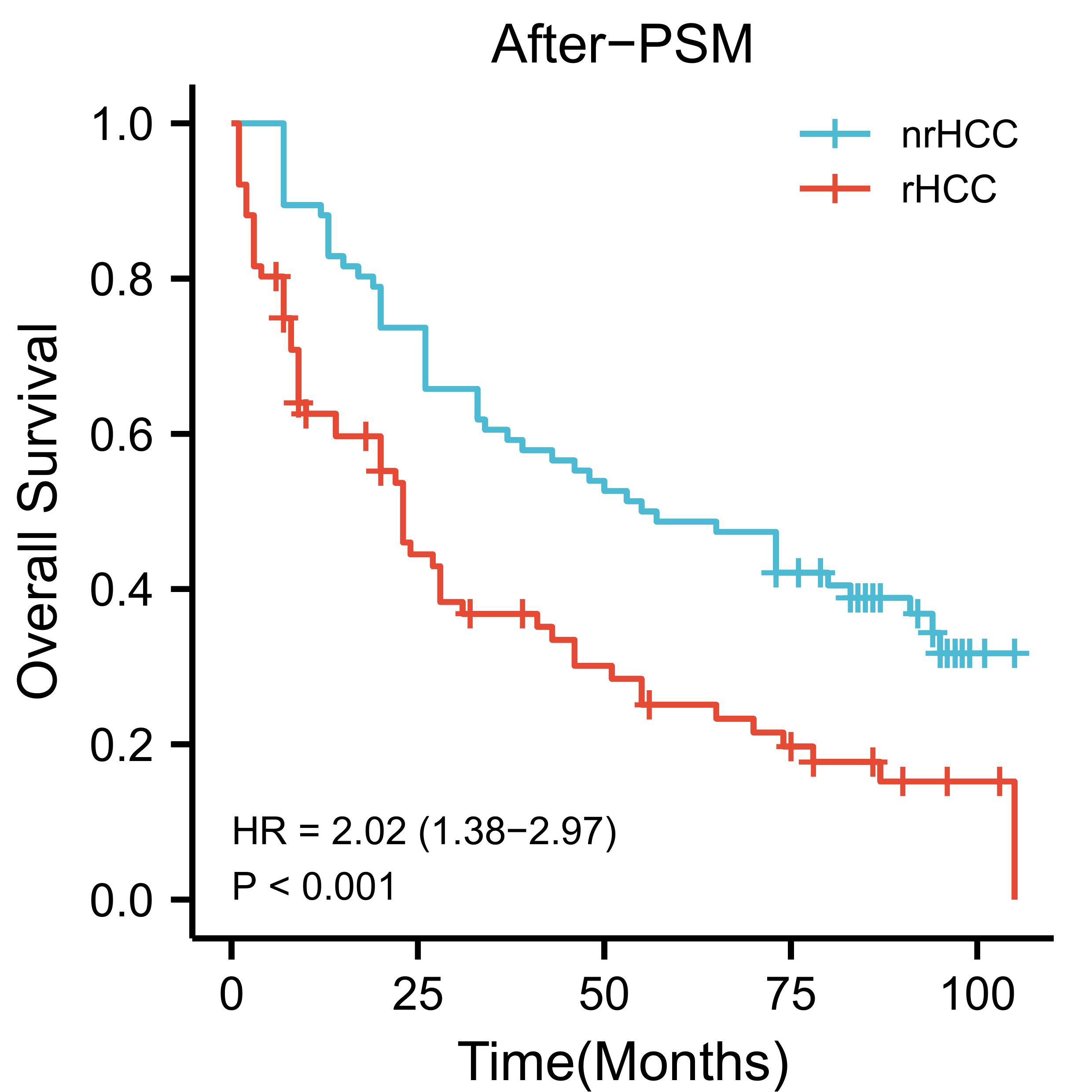

Supplement: Supplementary Figure 3 — Survival curves for overall survival in the nrHCC versus rHCC groups after PSM. [file Image_3.jpg]
