# Supplementary material for: Early-Stage Ruptured Hepatocellular Carcinoma With Different Tumor Diameters: Small Tumors Have a Better Prognosis
Source: Front Oncol. 2022 May 17;12:865696. doi: 10.3389/fonc.2022.865696 (PMC9152538; doi:10.3389/fonc.2022.865696)
Supplement: Supplementary file 4 [file Table_1.docx]

Supplementary Table 1. Baseline characteristics of nrHCC and rHCC patients in BCLC 0/A stage after 1:1 PSM. (n = 152)

|  |  | **rHCC (n=76)** | **nrHCC (n=76)** | **p-value** |
| --- | --- | --- | --- | --- |
|  |  |  |  |  |
| Gender |  |  |  | 0.564 |
|  | Male | 71(93.4) | 68(89.5) |  |
|  | Female | 5(6.6) | 8(10.5) |  |
| Age |  |  |  | 0.079 |
|  | ≤60 y | 59(77.6) | 68(89.5) |  |
|  | >60 y | 17(22.4) | 8(10.5) |  |
| Tumor number |  |  |  | 0.497 |
|  | Single | 74(97.4) | 76(100.0) |  |
|  | Multiple | 2(2.6) | 0(0.0) |  |
| AFP |  |  |  | 1.000 |
|  | ≤400ng/ml | 28(36.8) | 28(36.8) |  |
|  | >400ng/ml | 48(63.2) | 48(63.2) |  |
| Child-Pugh |  |  |  | 0.173 |
|  | A | 54(71.1) | 45(59.2) |  |
|  | B | 22(28.9) | 31(40.8) |  |
| Cirrhosis |  |  |  | 0.322 |
|  | No | 66(86.8) | 64(84.0) |  |
|  | Yes | 10(13.2) | 12(15.8) |  |
| HBsAg |  |  |  | 1.000 |
|  | No | 10(13.2) | 10(13.2) |  |
|  | Yes | 66(86.8) | 66(86.8) |  |
| ALB |  |  |  | 0.858 |
|  | ≤35g/L | 53(69.7) | 55(72.4) |  |
|  | >35g/L | 23(30.3) | 21(27.6) |  |
| ALT |  |  |  | 1.000 |
|  | ≤50U/L | 61(80.3) | 62(81.6) |  |
|  | >50U/L | 15(19.7) | 14(18.4) |  |
| AST |  |  |  | 0.867 |
|  | ≤40U/L | 49(64.5) | 47(61.8) |  |
|  | >40U/L | 27(35.5) | 29(38.2) |  |
| ALP |  |  |  | 0.694 |
|  | ≤100U/L | 60(80.0) | 58(76.3) |  |
|  | >100U/L | 15(20.0) | 18(23.7) |  |
| GGT |  |  |  | 0.143 |
|  | ≤60U/L | 46(60.5) | 36(47.4) |  |
|  | >60U/L | 30(39.5) | 40(52.6) |  |
| Main treatment |  |  |  | 0.678 |
|  | Hepatectomy | 52(68.4) | 49(64.5) |  |
|  | TACE | 22(28.9) | 23(30.3) |  |
|  | Conservative | 2(2.6) | 4(5.3) |  |

PSM: Propensity Matching Score;BCLC:Barcelona Clinic Liver Cancer;AFP:alpha-fetoprotein;HCC:hepatocellular carcinoma;rHCC:ruptured hepatocellular carcinoma;TACE:transcatheter arterial chemoembolization;HBsAg: hepatitis B surface antigen;ALB: albumin;ALT:Alanine aminotransferase;AST:Aspartate aminotransferase;ALP: alkaline phosphatase;GGT: γ-glutamyl transpeptidase
